# Cold storage characteristics of hardy kiwifruit, *Actinidia arguta* ‘Autumn Sense’: comparison between two cold storage temperatures

**DOI:** 10.3389/fpls.2025.1692735

**Published:** 2025-10-22

**Authors:** Uk Lee, Hyun Ji Eo, Chung Ryul Jung, Yonghyun Kim

**Affiliations:** Special Forest Resources Division, National Institute of Forest Science, Suwon, Republic of Korea

**Keywords:** hardy kiwifruit, cold storage, chilling injury, antioxidants, carbohydrate

## Abstract

Postharvest handling of hardy kiwifruit generally involves cold storage to prolong shelf life by delaying the ripening process. However, extended storage at near-freezing temperatures often results in undesirable chilling injury. To clarify the specific cold responses associated with the mitigation of chilling injury symptoms, we examined the physiological and molecular responses of hardy kiwifruit stored at very low (VL; 1°C) and moderate low (ML; 5°C) conditions. Fruits stored at VL conditions exhibited significantly delayed ripening. However, chilling injury symptoms such as pitting became increasingly severe during the mid to late phase of storage compared with that under ML conditions, indicating that VL conditions are more likely to induce chilling injury. Antioxidant activity in fruit stored under VL conditions was higher than that in fruit stored under ML conditions during the first four weeks, although the ascorbic acid content in VL-stored fruit steadily declined and remained lower than that in fruit stored under ML conditions. RNA-sequencing analysis revealed that most unigenes associated with antioxidant activity and the detoxification system were upregulated under VL conditions compared with that under ML conditions. Additionally, although unigenes involved in starch degradation were highly expressed under ML conditions, a subset of genes related to sucrose biosynthesis was upregulated under VL conditions, which corresponded to relatively higher sucrose levels in the later stages of storage. Our findings suggest that the antioxidant system and specific soluble carbohydrate metabolism are sensitive to lower cold storage temperatures, and their activation appears to contribute to the mitigation of chilling injury symptoms during storage.

## 1 Introduction

Hardy kiwi (*Actinidia arguta*) is a deciduous vine belonging to the family Actinidiaceae. Its fruit, commonly called kiwiberry, mini kiwi, baby kiwi, or hardy kiwifruit, is typically small with smooth, thin, edible green skin, though red-skinned varieties also exist (Fisk et al., 2008; Huang, 2016). Owing to its cold and disease resistance and short growth cycle, hardy kiwi is well considered for ecological cultivation (Horák et al., 2019; Qiao et al., 2025). It is rich in vitamin C, polyphenols, flavonoids, and anthocyanins, which are linked to various health benefits, such as reduced oxidative stress, enhanced immune function, and potential prevention of chronic diseases (Ferguson and Ferguson, 2002; Huang, 2016; Latocha, 2017; Hu et al., 2024). Due to its appeal to both growers and consumers, hardy kiwi is now commercially cultivated in the United States, Chile, New Zealand, Australia, South Korea, China, and many European countries and the area under cultivation is steadily increasing (Latocha, 2017; Wang et al., 2025).

Hardy kiwifruit is a climacteric fruit and is typically harvested before it fully ripens on the vine, as its soft peel and pulp make it highly susceptible to damage during shipping (Strik and Hummer, 2006). These delicate fruit characteristics pose a significant challenge for preservation and distribution, leading to short storability—a major limitation in the commercial production of hardy kiwifruit. Hardy kiwifruit is generally stored at low temperatures to extend its shelf life. Under storage conditions of 0°C and 90–95% relative humidity, the storage life of hardy kiwifruit is approximately 1–2 months (Strik and Hummer, 2006; Fisk et al., 2008). However, while cold storage helps maintain the freshness of fruit, improper temperature control can cause chilling injury, such as peel pitting and browning, which degrade fruit quality and reduce marketability (Eo et al., 2024; Xiong et al., 2024a). Susceptibility to cold damage of hardy kiwifruit varies among cultivars (Kim et al., 2020; Eo et al., 2024; Park et al., 2025). Activation of the phenylpropanoid pathway and its metabolites was recently reported to be associated with reduced chilling injury in some cultivars of hardy kiwifruit (Park et al., 2025). Additionally, differences in antioxidant levels among cultivars may influence the extent of chilling injury (Eo et al., 2024). Meanwhile, recent studies have shown that supplementary treatments can be effective in reducing chilling injury in hardy kiwifruit during cold storage (Xiong et al., 2024a, b). These findings highlight that problem of chilling injury of hardy kiwifruit being a key issue under cold storage conditions.

Hardy kiwi cultivars are cold-tolerant plants with superior frost hardiness than that of fuzzy kiwi cultivars. However, frost tolerance does not necessarily translate into improved fruit storability under cold conditions. In fact, fuzzy kiwifruit cultivars such as ‘Hayward’ can maintain marketable quality for over four months in cold storage at temperatures near 0°C. To develop and implement effective cold storage techniques for hardy kiwifruit, an understanding of the precise mechanisms underlying the cold response in this fruit is essential. This is because the development of new postharvest technologies may require both broadly applicable and cultivar-specific strategies, depending on the characteristics of fruit species and varieties. Therefore, to clarify the specific cold responses associated with the mitigation of chilling injury symptoms, we hypothesized that hardy kiwifruit would exhibit distinct cold responses depending on storage temperature. To test this hypothesis, we investigated the cultivar ‘Autumn Sense’ by evaluating its physiological properties and conducting transcriptomic analyses under two storage conditions: very low (VL; 1°C) and moderately low (ML; 5°C). This study provides valuable insights for the development and improvement of postharvest techniques and technologies for hardy kiwifruit. Our findings should contribute to future research on cultivar breeding, fruit storage behavior, and cold storage potential.

## 2 Materials and methods

### 2.1 Plant material

Fruits of the ‘Autumn Sense’ cultivar were harvested at commercial maturity stage, defined by a soluble solids content of 7–8%, between 105 and 110 days after full bloom, from five-year-old trees cultivated in a hardy kiwifruit orchard located in Wonju, Gangwon-do, Republic of Korea. Immediately after harvest, the fruits were transported to a cold storage facility at the National Institute of Forest Science in Suwon, Gyeonggi-do, Republic of Korea. Only uniform, undamaged fruits were selected for the experimentation. The fruits were packed in perforated polyethylene terephthalate (PET) boxes, each containing approximately 22–25 fruits (500–520 g). The twelve PET boxes were placed in plastic containers. The containers were stored under controlled conditions at two temperatures—1°C (very low, VL) and 5°C (moderate low, ML)—with relative humidity maintained at approximately 85–90% for 8 weeks. Each storage condition included three plastic containers (36 PET boxes in total, containing approximately 18–20 kg of fruit per treatment). Fresh fruits were sampled from both storage temperatures at two-week intervals and either used to evaluate quality attributes or rapidly frozen in liquid nitrogen and stored at –80°C for subsequent molecular and biochemical analyses.

### 2.2 Evaluation of quality attributes

The rate of weight loss was determined by weighing PET boxes containing fruit samples at 2-week intervals throughout the storage period, in triplicate. Weight loss was expressed as a percentage of initial weight at each sampling interval. Fruit firmness was measured using a texture analyzer (CT3; AMETEK Brookfield, Middleboro, MA, USA) equipped with a flat probe (diameter, 2 mm), operated at a compression speed of 1 mm/s, with 17–20 biological replicates. The probe was applied to the center of the fruit’s flat surface and compressed to a depth of 10 mm. The results were expressed in Newtons (N). Total soluble solids content (TSSC; %) was examined using a digital refractometer (PR-101a; ATAGO, Tokyo, Japan) with 17–20 biological replicates. Titratable acidity (TA; %) was determined using a Titrator EasyPlus Easy pH system (Mettler Toledo, Columbus, OH, USA) with 10 biological replicates. The results were expressed as a percentage of anhydrous citric acid. The incidence of shrinking, browning, pitting, and decay was assessed using a 6-point scale based on the percentage of the fruit surface exhibiting each symptom: 0 = 0%; 1 = 1–20%; 2 = 21–40%; 3 = 41–60%; 4 = 61–80%; and 5 = 81–100% of the fruit surface area affected, in 17–20 biological replicates.

### 2.3 Color measurement

Color characteristics were assessed using a Minolta Chroma Meter (Model CR-400; Konica Minolta Optics, Osaka, Japan) on 10–20 randomly selected hardy kiwifruits per treatment. Measurements were taken at three equidistant points on the surface of each fruit. Color parameters were expressed using the Hunter scale (L* (lightness); a* (green to red); and b* (blue to yellow)) to compare the color differences between treatments.

### 2.4 Sample preparation for determination of the levels of metabolites

Whole hardy kiwifruits, including both peel and pulp, were freeze-dried using a freeze dryer (MG-VFD20; MG Industry, Gunpo, South Korea), and the dried samples were ground into a fine powder using a laboratory grinder (IKA Multidrive Basic; IKA Korea, Seoul, South Korea). The powdered material was used for subsequent analyses.

### 2.5 Determination of the total chlorophyll, carotenoid, flavonoid, and phenolic content

To extract total chlorophylls, carotenoids, and flavonoids, 0.2 g of the freeze-dried powdered sample was homogenized in 20 mL of 80% acetone. The mixture was filtered through a No. 51 filter paper (Hyundai Micro, Seoul, Republic of Korea), and the filtrate was used for subsequent analyses. The concentrations of total chlorophylls and carotenoids in the filtered extracts were quantified using a spectrophotometer (Epoch 2; Agilent Technologies, Santa Clara, CA, USA) by measuring the absorbance at 662, 645, and 470 nm. The absorbance values were used to calculate the total chlorophyll and carotenoid concentrations according to the methods described by Arnon (1949) and Win et al. (2021). Total flavonoids in the filtered extract were quantified using the method described by Zhishen et al. (1999) with slight modification. The filtered extract (1 mL), distilled water (4 mL), and 5% NaNO_2_ (0.3 mL) were mixed in a clean tube and incubated at 25°C for 5 min. Subsequently, AlCl_3_ (0.3 mL) was added to the mixture. The tube was vortexed and the mixture was incubated again at 25°C for 6 min. Subsequently, 2.4 mL of 1 M NaOH and 2.4 mL of distilled water were added to the tube, followed by thorough vortexing. The absorbance of the final solution was measured at 510 nm using a spectrophotometer (Epoch 2). The concentration of total flavonoids was determined by comparing the absorbance of the samples to a standard curve generated using catechin standards (Sigma-Aldrich, St. Louis, MO, USA). To extract total phenolics, freeze-dried powdered sample (0.2 g) was homogenized in 20 mL of methanol containing 1% hydrochloric acid (MeOH: HCl = 99:1, v/v). The homogenate was centrifuged at 3,000 rpm for 20 min at 10°C. The supernatant was used to measure the amount of total phenolics using the Folin–Ciocalteu method (Singleton et al., 1999). Briefly, the supernatant (0.1 mL), MeOH (0.1 mL), and the Folin–Ciocalteu reagent (0.1 mL) were mixed in a clean tube and incubated for 6 min in the dark at 25°C. Thereafter, 20% Na_2_CO_3_ (0.7 mL) was added to the mixture and vortexed. The resulting mixture was incubated in the dark at 25°C for 60 min and centrifuged at 13,500 rpm for 3 min at 4°C. The absorbance of the supernatant was measured at 735 nm using a spectrophotometer (Epoch 2). A standard curve generated using gallic acid (Sigma-Aldrich) was used to determine the total phenolic concentration. The experiments in this section were performed in triplicate, using fine powder obtained from ten freeze-dried fruits per replicate.

### 2.6 Measurement of antioxidant activity

Antioxidants were extracted from freeze-dried powdered samples (0.2 g), as described previously (Eo et al., 2024). The 2,2-diphenyl-1-picrylhydrazyl (DPPH)-scavenging activity of the extracts was measured as described by Stoilova et al. (2007), with slight modifications. Briefly, the extract (0.2 mL) was mixed with 1 mL of 0.4 mM DPPH solution and incubated at 25°C for 30 min in the dark. After incubation, the absorbance of the mixture was measured at 517 nm using a spectrophotometer (Epoch 2). The results were expressed as DPPH radical-scavenging activity (%). The 2,2′-Azino-bis (3-ethylbenzothiazoline-6-sulfonic acid (ABTS)-scavenging activity was determined as described by Biglari et al. (2008), with some modifications. Briefly, a mixture of 7 mM ABTS and 2.45 mM potassium persulfate was incubated in the dark at 25°C for 16 h. The resulting solution was diluted with 70% ethanol to achieve an absorbance of approximately 1.5 at 734 nm. The extract was then added to the diluted ABTS solution and incubated at 25°C for an additional 6 min. The absorbance was immediately measured at 734 nm using a spectrophotometer (Epoch 2) and converted to ABTS-scavenging activity (%). Antioxidant activity measurements were performed in triplicate, using extracts prepared from fine powder obtained from ten freeze-dried fruits per replicate.

### 2.7 Determination of the ascorbic acid content

Ascorbic acid (AsA) was extracted from freeze-dried powdered sample (0.2 g), following a previously described method (Eo et al., 2024). The extract was filtered through a 0.45 μm syringe filter and subsequently analyzed for AsA concentration using an Ultimate 3000 ultra-high-performance liquid chromatography (UHPLC) system (Thermo Fisher Scientific, Waltham, MA, USA). The UHPLC conditions were as described previously (Eo et al., 2024). The concentration of AsA in the extract was determined using a calibration curve generated from an authentic AsA standard (Sigma-Aldrich). AsA content was determined in triplicate using extracts prepared from fine powder obtained from ten freeze-dried fruits per replicate.

### 2.8 Determination of lipid peroxidation

The level of lipid peroxidation was measured using a previously described method, with minor modifications (Metwally et al., 2003). The freeze-dried powdered sample (0.2 g) was homogenized in 10 mL of 0.1% (w/v) trichloroacetic acid, followed by centrifugation at 3,000 rpm for 10 min at 25°C. A 1 mL aliquot of the resulting supernatant was mixed with 4 mL of a solution containing 20% (w/v) trichloroacetic acid and 0.5% (w/v) thiobarbituric acid. The mixture was incubated in a water bath at 95°C for 15 min and then immediately cooled on ice. The absorbance was measured at 532 nm using a spectrophotometer (Epoch 2). To correct for nonspecific absorbance, the reading at 600 nm was subtracted from that at 532 nm. The concentration of malondialdehyde (MDA) was calculated using an extinction coefficient of 155 mmol^–1^ L^–1^ cm^–1^, with three biological replicates. The determination of MDA content was performed in triplicate, using extracts prepared from fine powder obtained from ten freeze-dried fruits per replicate.

### 2.9 Total RNA isolation

Total RNA was isolated from hardy kiwifruit using the RNeasy Plant Mini Kit (Qiagen, Hilden, Germany), following the manufacturer’s protocol with slight modifications. Briefly, frozen, hardy kiwifruit was finely ground in liquid nitrogen using a mortar and pestle. The resulting powder was lysed in 450 μL of RNeasy RLT buffer containing 3% beta-mercaptoethanol (v/v). The lysate was homogenized by passing through a QIAshredder spin column at 20,000 × *g* for 2 min. The flow-through was collected and mixed with 225 μL of 99.5% ethanol. This mixture was loaded onto an RNeasy spin column and centrifuged at 10,000 × *g* for 1 min. The spin column was subsequently washed with 350 μL of RW1 buffer (Qiagen) under the same centrifugation conditions. The DNase treatment was performed by applying RNase-free DNase (Qiagen) directly onto the spin column, followed by incubation at room temperature for 15 min. The spin column was washed with 350 μL of RW1 buffer and 500 μL of RPE buffer, centrifuging for 1 min at 10,000 × *g* after each wash, and then subjected to an additional wash with 500 μL of RPE buffer for 2 min at the same speed. To remove the residual ethanol, the spin column was transferred to a clean collection tube and centrifuged at 15,000 × *g* for 1 min. Total RNA was eluted by applying 30 μL of nuclease-free water directly onto the column membrane, followed by centrifugation at 10,000 × *g* for 1 min. The eluted total RNA was stored at −80°C. RNA concentration and purity were assessed spectrophotometrically at 260 and 280 nm using a NanoDrop One (Thermo Fisher Scientific, Waltham, MA). RNA integrity was evaluated using an Agilent 2100 Bioanalyzer with an RNA 6000 Pico kit (Agilent Technologies, Santa Clara, CA), and samples with RNA integrity number greater than 8 were used for further analyses.

### 2.10 Library construction and sequencing

RNA library construction and sequencing were conducted using total RNA extracted from hardy kiwifruit, with three biological replicates per treatment. Libraries were prepared from the total RNA using the TruSeq Small RNA Library Prep kit (Illumina, San Diego, CA), following the manufacturer’s instructions. Sequencing of the libraries was carried out on an Illumina NovaSeq 6000 platform (Illumina, San Diego, CA, USA) using 100-basepair paired-end reads for RNA-sequencing (RNA-seq) analysis, which was conducted by Macrogen (Seoul, Republic of Korea).

### 2.11 *De novo* RNA-seq data processing and analysis

Low-quality reads, adapter sequences, contaminant DNA, and PCR duplicates were removed from the raw sequencing data to reduce bias in downstream analyses. High-quality reads from all samples were merged into a single dataset for transcriptome assembly. *De novo* transcriptome reconstruction was performed using Trinity (version trinityrnaseq_r20140717; Bowtie version 1.1.2; https://github.com/trinityrnaseq/trinityrnaseq/wiki), which assembles overlapping reads into contiguous sequences (contigs) without introducing N gaps. The longest contig was retained for each locus. The assembled contigs were filtered and clustered into non-redundant transcripts using CD-HIT-EST (version 4.6; http://weizhongli-lab.org/cd-hit), and the resulting representative sequences were defined as unigenes. The unigenes were subjected to functional annotation by querying against several public databases, including the NCBI Nucleotide (NT), NCBI non-redundant protein (NR), UniProt, Kyoto encyclopedia of genes and genomes (KEGG), Pfam, gene ontology (GO), and EggNOG. Sequence similarity searches were performed using BLASTN (NCBI BLAST version 2.9.0+; https://blast.ncbi.nlm.nih.gov/Blast.cgi) and BLASTX via DIAMOND (version 0.9.21; https://github.com/bbuchfink/diamond). Transcript abundance was estimated using the RSEM algorithm (version v1.3.1; Bowtie version 1.1.2; http://deweylab.github.io/RSEM). The resulting read counts were normalized across samples using the Relative Log Expression (RLE) method, and gene expression levels were quantified as normalized read counts. Differentially expressed genes (DEGs) were identified based on an absolute log2 fold change ≥ 1 and a false discovery rate (FDR) < 0.05 between VL and ML conditions (*n* = 3). For functional classification of the unigenes, GO annotation was performed using DIAMOND-based BLASTX searches, and the resulting GO terms were categorized into biological process (BP), cellular component (CC), and molecular function (MF) categories (**Supplementary Figure S1**, in the **Supplementary Materials**). All RNA-seq data were deposited in the NCBI Sequence Read Archive (SRA) under the BioProject accession number PRJNA1028359 (https://www.ncbi.nlm.nih.gov/sra/?term=PRJNA1028359).

### 2.12 Gene expression analysis

Gene expression analysis was performed using reverse transcription-quantitative polymerase chain reaction (RT-qPCR) with 3–4 biological replicates. Template cDNA was synthesized from 250 ng of total RNA using the PrimeScript RT reagent kit with a gDNA eraser (Takara Bio, Shiga, Japan), following the manufacturer’s instructions. RT-qPCR was conducted using a CFX96 Touch Real-Time PCR Detection System (Bio-Rad, Hercules, CA, USA) with iQ™ SYBR^®^ Green Supermix (Bio-Rad) in a total reaction volume of 20 μL, which included 2 μL of cDNA template. Relative gene expression levels were calculated using the comparative CT method (ΔΔCT) as described by Schmittgen and Livak (2008). *AaActin* was used as an internal reference gene for normalization (Lin et al., 2022). Primer sequences used in this study are listed in **Supplementary Table S1** in the **Supplementary Materials**.

### 2.13 Estimation of the starch and soluble sugar content

Starch and soluble sugars were extracted from freeze-dried powdered sample (0.1 g), according to a previously described method (Eo et al., 2024). Starch and sugars were quantified in triplicate using enzymatic assay kits for starch and for sucrose/D-glucose/D-fructose, respectively (R-Biopharm AG, Darmstadt, Germany), with extracts prepared from fine powder of ten freeze-dried fruits per replicate.

### 2.14 Statistical analysis

All data are expressed as the mean ± standard deviation (SD). Statistical significance between treatments at each time point was assessed using Student’s *t*-test in GraphPad Prism version 10 (GraphPad Software, San Diego, CA, USA).

## 3 Results

### 3.1 Changes in quality attributes of hardy kiwifruit during cold storage

**Figure 1:**
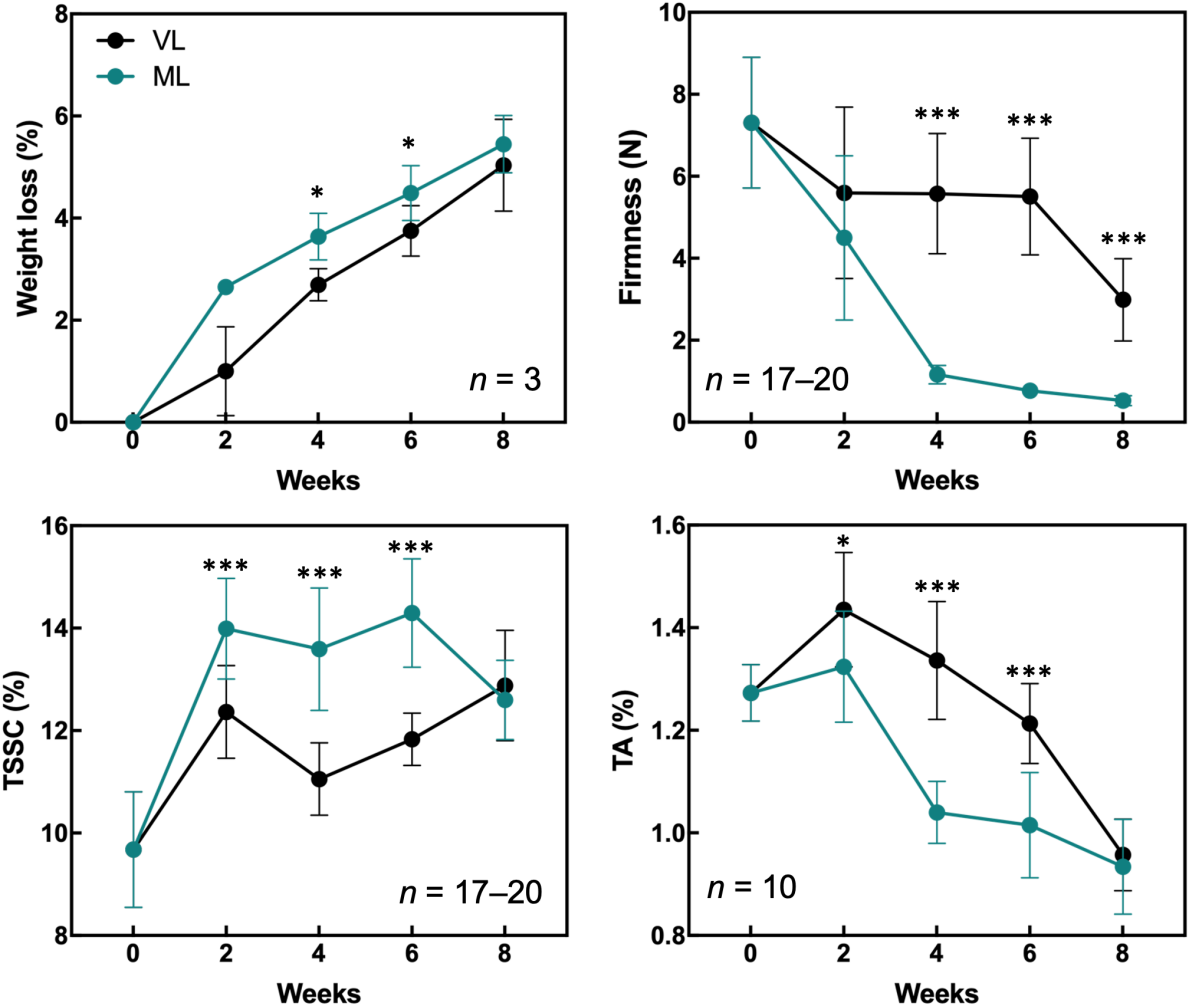
Quality attributes of hardy kiwifruit were evaluated during the 8-week cold storage period. The parameters measured included weight loss, firmness, total soluble solids content (TSSC), and titratable acidity (TA). Asterisks denote significant differences between the VL and ML conditions, as determined using the Student’s *t*-test (*p < 0.05 and ***p < 0.001).

**Figure 2:**
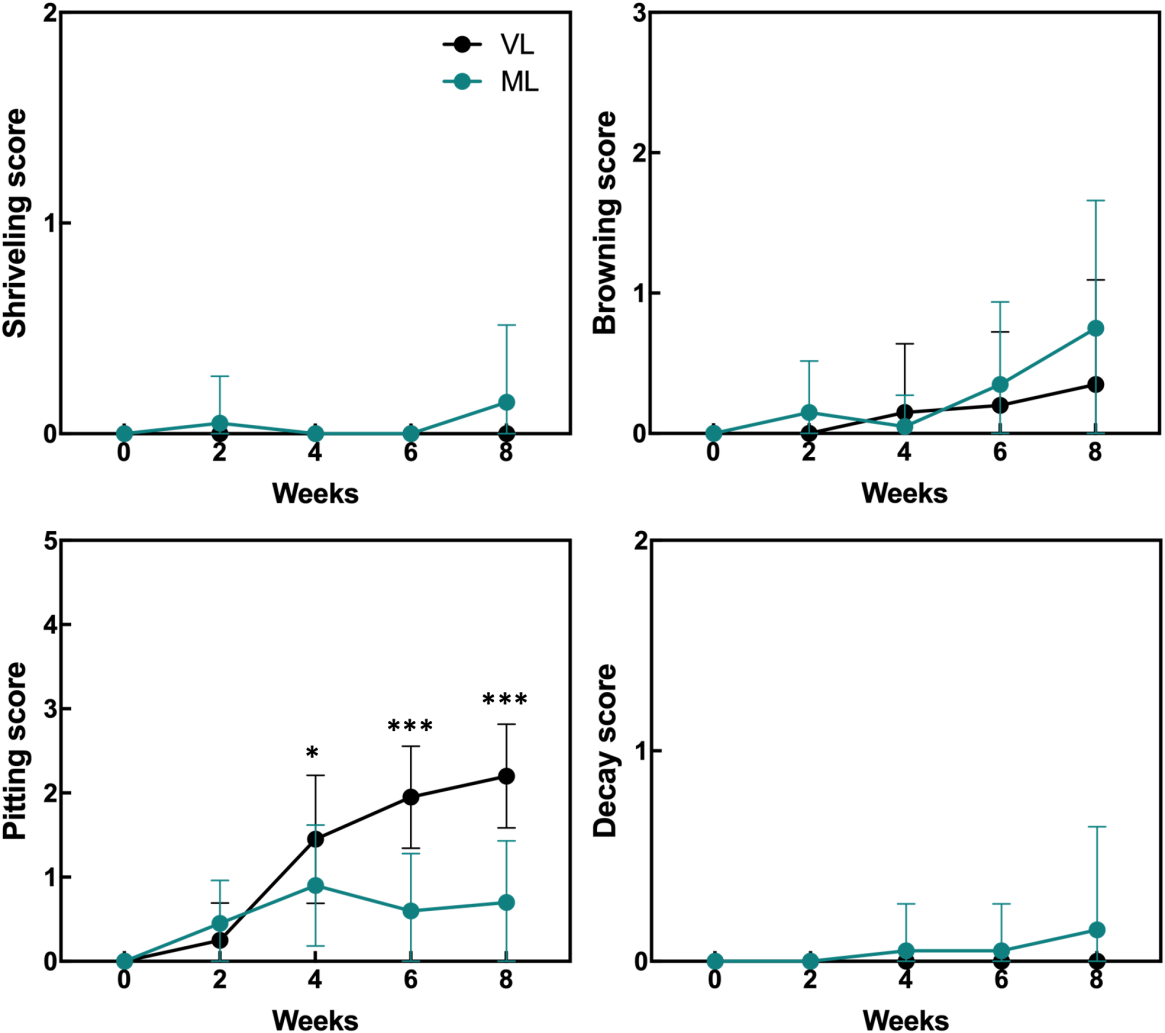
Incidence of physiological disorders in hardy kiwifruit during cold storage was assessed by evaluating shriveling, browning, pitting, and decay using a 6-point scale: 0 = 0%; 1 = 1–20%; 2 = 21–40%; 3 = 41–60%; 4 = 61–80%; and 5 = 81–100% of the fruit surface area affected. Asterisks denote significant differences between the VL and ML conditions, as determined using the Student’s *t*-test (*p < 0.05 and ***p < 0.001; *n* = 17–20).

**Figure 3:**
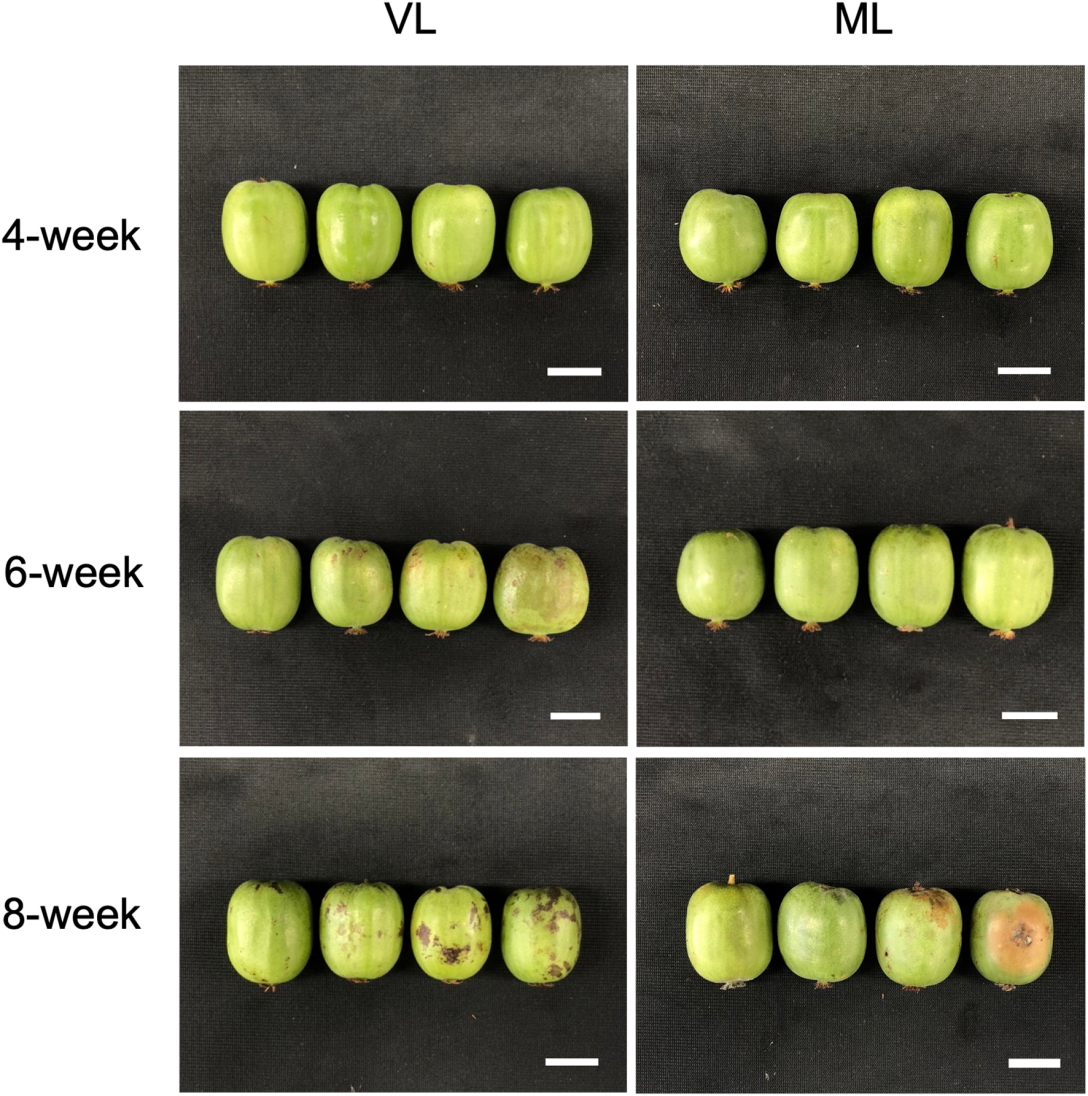
Hardy kiwifruit stored under VL and ML conditions for 8 weeks of storage. Scale bars = 2 cm.

**Figure 4:**
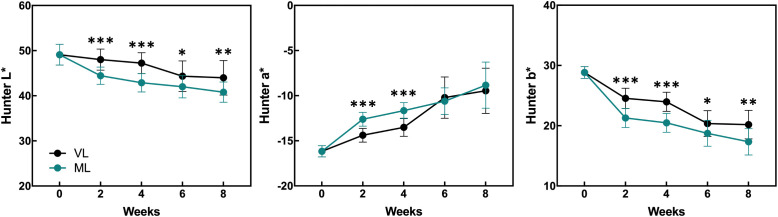
Changes in the Hunter values were monitored during the storage period and compared between the storage temperatures. Asterisks denote significant differences between the VL and ML conditions, as determined using the Student’s *t*-test (*p < 0.05, **p < 0.01, and ***p < 0.001; *n* = 10–20).

Hardy kiwifruit exhibits climacteric behavior, with both internal and external tissues gradually ripening during cold storage. At both the storage temperatures, fruit weight loss steadily increased over the 8-week period (**Figure 1**). However, the extent of weight loss was significantly lower in the VL group than in the ML group. Changes in fruit firmness, TSSC, and TA are key indicators of fruit ripening; typically, TSSC increases, while both firmness and TA decrease as ripening progresses, directly influencing the overall shelf life of hardy kiwifruit. In the present study, fruit firmness was better maintained in the VL group for up to 6 weeks, remaining significantly higher than in the ML group throughout storage. In the ML group, TSSC increased rapidly during the first two weeks, stayed significantly higher than in the VL group up to 6 weeks, and then declined by week 8. TA decreased in both groups, with a significantly greater reduction observed in the ML group compared to the VL group. Overall, these results indicate that fruit ripening progressed more rapidly under ML conditions than under VL conditions. The incidence of physiological disorders of the hardy kiwifruit also varied between the two groups (**Figure 2**). No significant differences were observed between the treatments in terms of shriveling, browning, and decay scores, although fruits in the ML group tended to show higher browning and decay than those in the VL group over the period of storage. However, pitting scores differed significantly between the two treatments, with fruits in the VL group exhibiting markedly higher pitting, a major symptom of chilling injury, after 2 weeks (**Figures 2**, **3**). These results suggest that specific cold injury symptoms occurred more frequently as the cold storage temperature decreased. Color characteristics, as measured using Hunter parameters, also varied between the storage conditions (**Figure 4**). Hunter L (lightness) and b (yellowness) values decreased over time, with VL samples maintaining significantly higher values than those stored under ML conditions. In contrast, Hunter a (redness) was higher in the ML group for up to 4 weeks than in the VL group. These results implied that fruits stored under VL conditions retained a fresher appearance and underwent slower ripening than those stored under ML conditions, although they exhibited greater susceptibility to chilling injury.

### 3.2 Changes in total chlorophyll, carotenoid, flavonoid, phenolic, ascorbic acid, and malondialdehyde content and antioxidant activity

**Figure 5:**
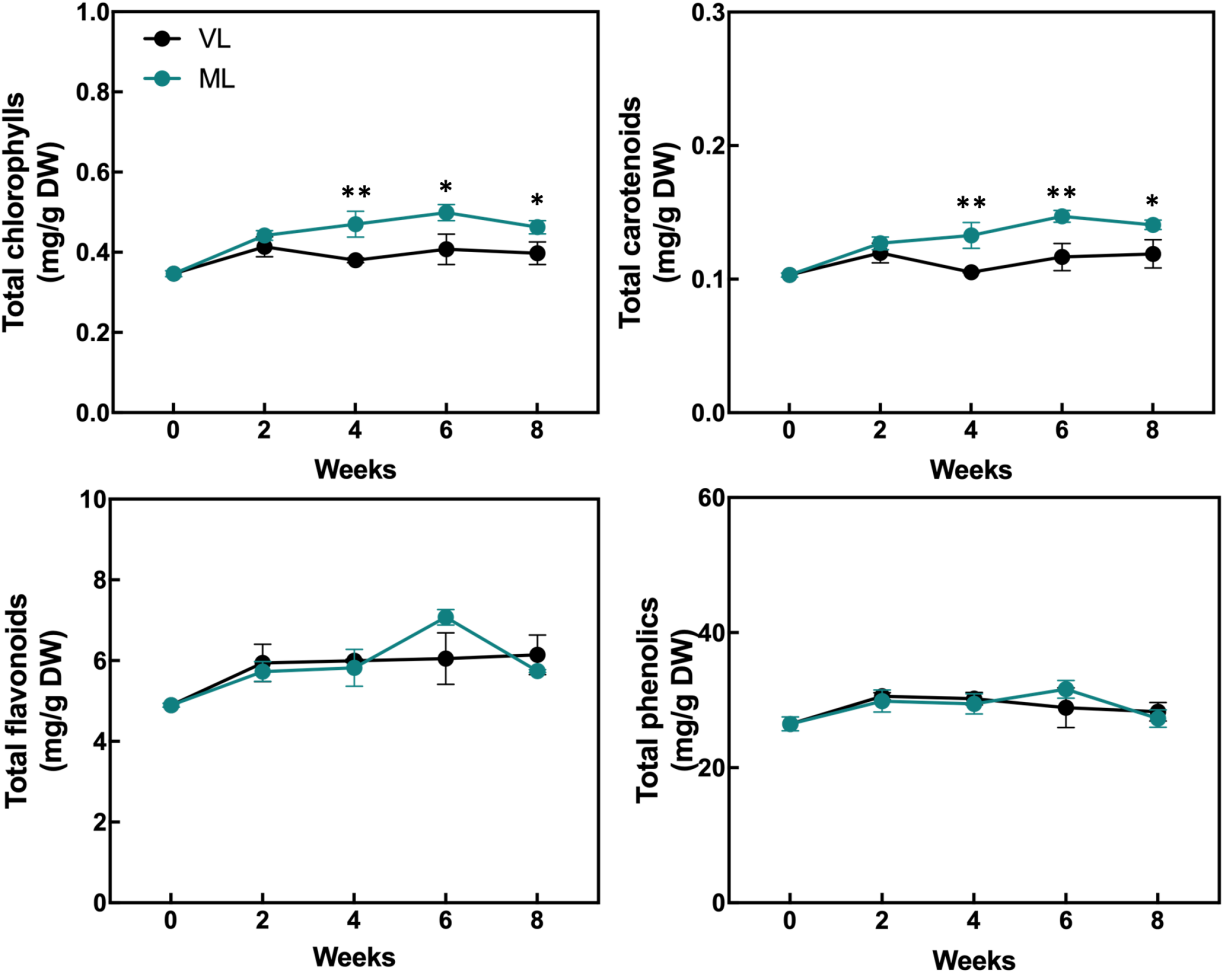
Changes in the content of total chlorophylls, carotenoids, flavonoids, and phenolics were assessed. Asterisks denote significant differences between the VL and ML conditions, as determined using the Student’s *t*-test (*p < 0.05 and **p < 0.01; *n* = 3).

**Figure 6:**
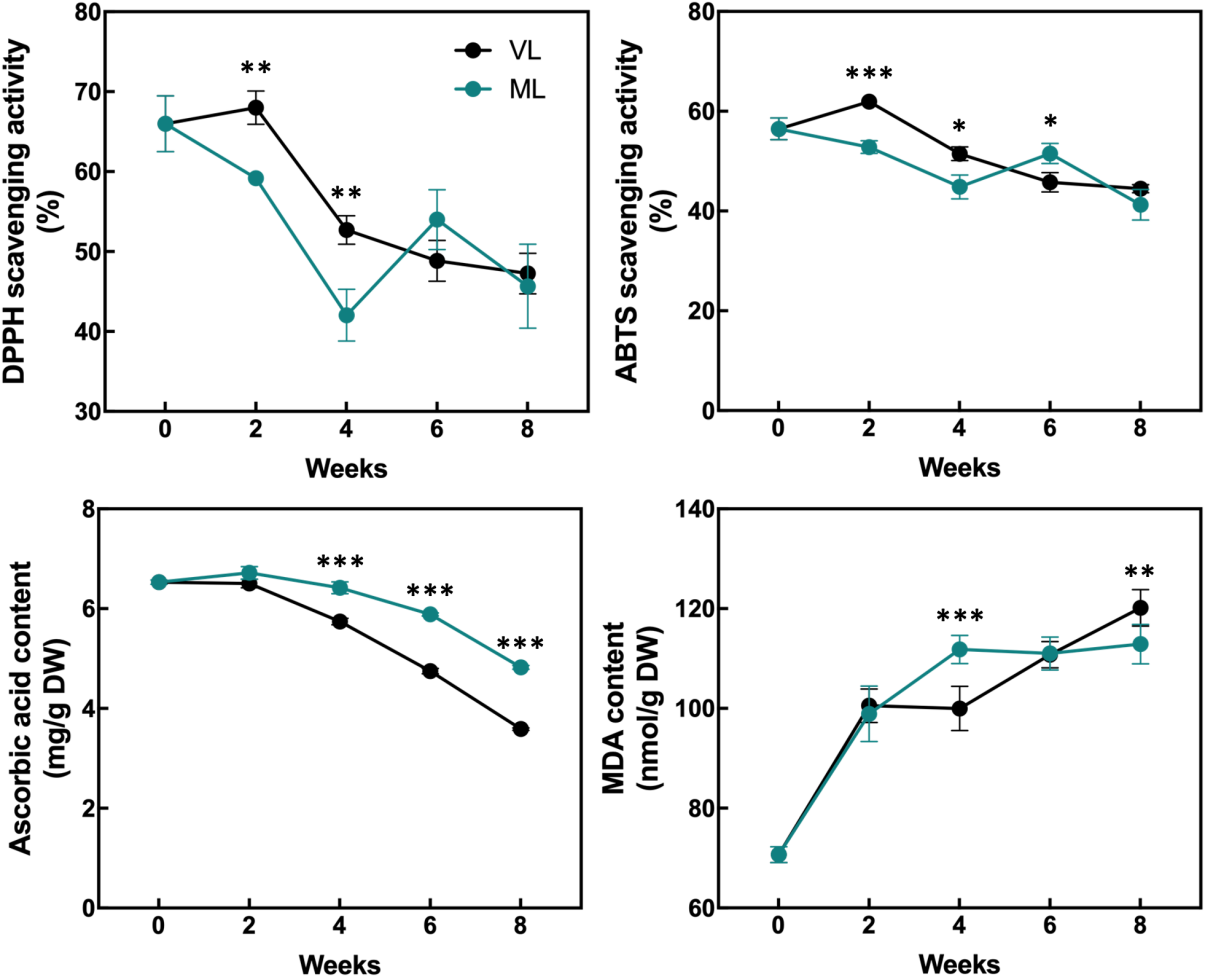
Changes in the antioxidant activities, ascorbic acid content, and malondialdehyde (MDA) content were assessed. Asterisks denote significant differences between the VL and ML conditions, as determined using the Student’s *t*-test (*p < 0.05, **p < 0.01, and ***p < 0.001; *n* = 3).

The levels of total chlorophylls and carotenoids were maintained throughout the storage period in both the ML and VL groups, although the ML group exhibited slightly higher levels of total chlorophylls and carotenoids after 2 weeks, with statistically significant differences (**Figure 5**). The levels of total flavonoids and phenolics were generally preserved in both the treatments and no significant differences were observed throughout the storage period. The DPPH radical scavenging activity was significantly higher in the VL group fruit than in the ML group ones up to 4 weeks (**Figure 6**), although the activity in the VL group declined after 2 weeks, and similar levels were observed between treatments from week 6. The ABTS scavenging activity showed a similar overall trend, although ML exhibited significantly higher activity at week 6. Interestingly, the level of AsA, a key health-promoting compound in hardy kiwifruit, was consistently and significantly higher in the ML group fruits than in the VL group fruits throughout the storage period. Additionally, the MDA content, an indicator of lipid peroxidation and oxidative stress, was significantly higher in the ML group at 4 weeks, but increased continuously in the VL group up to 8 weeks. Although storage under VL conditions effectively preserved chlorophylls, carotenoids, flavonoids, and phenolics and maintained higher antioxidant activity during early storage, it had a detrimental effect on the AsA content. Based on these results, AsA appears to serve as a major antioxidant, potentially alleviating oxidative damage induced by chilling injury under cold storage conditions. This hypothesis is further supported by the progressive accumulation of MDA in fruits stored under the VL conditions, with significantly higher levels detected at 8 weeks compared with that under ML conditions, indicating more severe oxidative stress over time.

### 3.3 Changes in the expression of genes related to antioxidant and detoxification systems, including ascorbic acid biosynthesis and regeneration pathway

**Figure 7:**
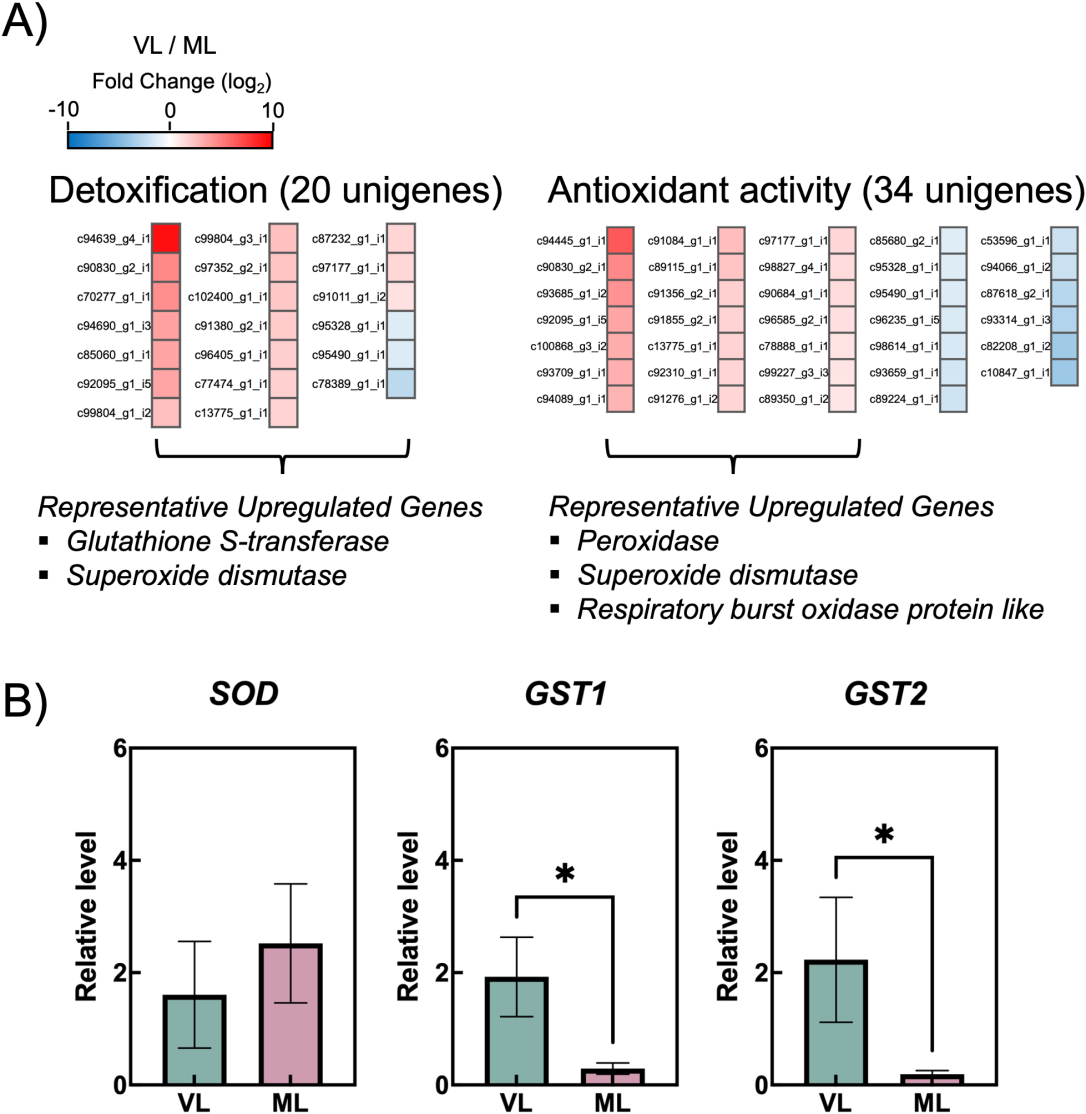
**(A)** Heatmap showing differentially expressed genes (colored squares) with an absolute value of log_2_ fold change (FC) no less than 1 (*p* < 0.05) and false discovery rate (FDR) less than 0.05 in two gene ontology (GO) terms: “Detoxification” and “antioxidant activity.” **(B)** Relative transcript levels of contigs coding for superoxide dismutase (SOD) and glut athione S-transferases (GSTs), analyzed via RT-qPCR. Asterisks indicate significant differences between the VL and ML conditions, according to the Student’s *t*-test (*p < 0.05; *n* = 3–4).

Chilling injury in fruits under cold storage is attributed to oxidative stress caused by an imbalance between reactive oxygen species (ROS) production and scavenging. While ROS are a major contributor to chilling injury in cold-stored fruits, they also play a dual role by triggering antioxidant defenses that mitigate oxidative stress (Wang et al., 2013). To investigate the effects of cold storage conditions on changes in antioxidant mechanisms, *de novo* RNA-seq analysis was performed using hardy kiwifruit samples stored for 4 weeks. This time point was selected because it marks the initial onset of a pronounced difference in chilling injury between the VL and ML storage conditions (**Figure 2**). Of the 107,925 assembled unigenes, 40,591 were successfully annotated in at least one public database. A total of 7,992 DEGs were identified, including 3,747 upregulated and 4,245 downregulated unigenes, in VL compared with those in ML. DEGs were functionally annotated using GO terms via BLASTX analysis using the DIAMOND software and classified into 26 BP (24.37%), 14 CC (26.66%), and 13 MF (24.92%) (**Supplementary Figure S1**). Notably, the GO terms related to detoxification (0.07%) and antioxidant activity (0.17%) were categorized under BP and MF, respectively. Among the 20 unigenes classified under detoxification, including members of the *glutathione S-transferase* (*GST*) and *superoxide dismutase* (*SOD*) families, most were upregulated in the VL group (**Figure 7A**; **Supplementary Tables S2**, **S3**). Similarly, the unigenes associated with antioxidant activity, such as those encoding peroxidase (POD) and SOD, were predominantly upregulated under VL conditions. The selected highly upregulated detoxification unigenes (log_2_ FC > 4), *SOD*, *GST1* and *GST2*, were validated using RT-qPCR. Notably, two *GSTs* were significantly upregulated in the VL group compared with those in the ML group (**Figure 7B**).

**Figure 8:**
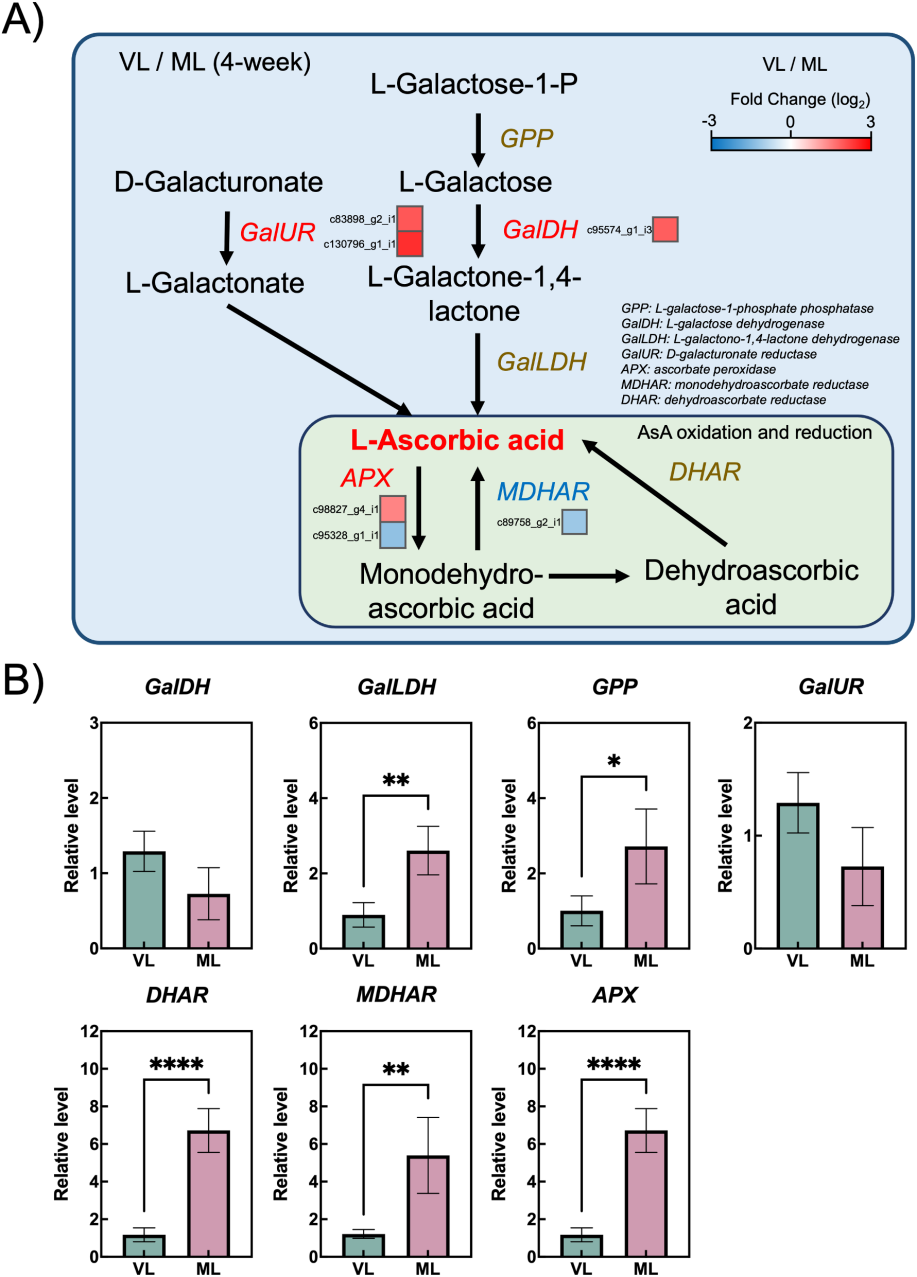
Ascorbic biosynthesis and regeneration pathway. **(A)** Differentially expressed genes (colored squares below or to the right of each gene) with absolute log_2_ fold change (|log_2_ FC| ≥ 1), p < 0.05, and false discovery rate (FDR) < 0.05. **(B)** Relative transcript levels of ascorbic acid biosynthesis and regeneration -related genes analyzed via RT-qPCR. Asterisks indicate significant differences between the VL and ML conditions, according to the Student’s *t*-test (*p < 0.05, **p < 0.01, and ****p < 0.0001; *n* = 3–4).

The AsA biosynthetic and regeneration pathways are key components of the antioxidant system and play an essential role in chilling tolerance in kiwifruit (Liu et al., 2023). To examine the molecular differences between the VL and ML conditions, DEGs involved in AsA metabolism were analyzed. Among the identified DEGs, only two genes encoding L-galactose dehydrogenase (GalDH) and D-galacturonate reductase (GalUR) were upregulated under VL conditions, being involved in the L-galactose pathway and an alternative biosynthetic pathway, respectively (Wheeler et al., 2015) (**Figure 8A**; **Supplementary Table S4**). Meanwhile, genes involved in the AsA regeneration pathway, such as *ascorbate peroxidase* (*APX*) and *monodehydroascorbate reductase* (*MDHAR*), showed variable expression between the two conditions. To investigate the precise response of the AsA biosynthetic and regeneration pathways, the relative expression levels of related genes were further examined by RT-qPCR using primers previously reported by Lin et al. (2022). Although the expression levels of *GalDH* and *GalUR* were higher in the VL group, the differences were not statistically significant (**Figure 8B**). In contrast, *L-galactono-1,4-lactone dehydrogenase* (*GalLDH*) and *L-galactose-1-phosphate phosphatase* (*GPP*) were highly expressed in the ML group, implying that this branch of the AsA biosynthetic pathway is more active under ML conditions, consistent with the higher AsA levels observed in ML fruits (**Figure 6**). Moreover, the expression levels of *dehydroascorbate reductase* (*DHAR*), *MDHAR*, and *APX* were generally higher in ML samples, indicating that the AsA regeneration pathway was less active under VL conditions. These results suggest that the coordinated activation of both the AsA biosynthetic and regeneration pathways is associated with the maintenance of AsA levels in hardy kiwifruit during cold storage.

### 3.4 Changes in the expression of genes related to starch degradation and carbohydrate levels

**Figure 9:**
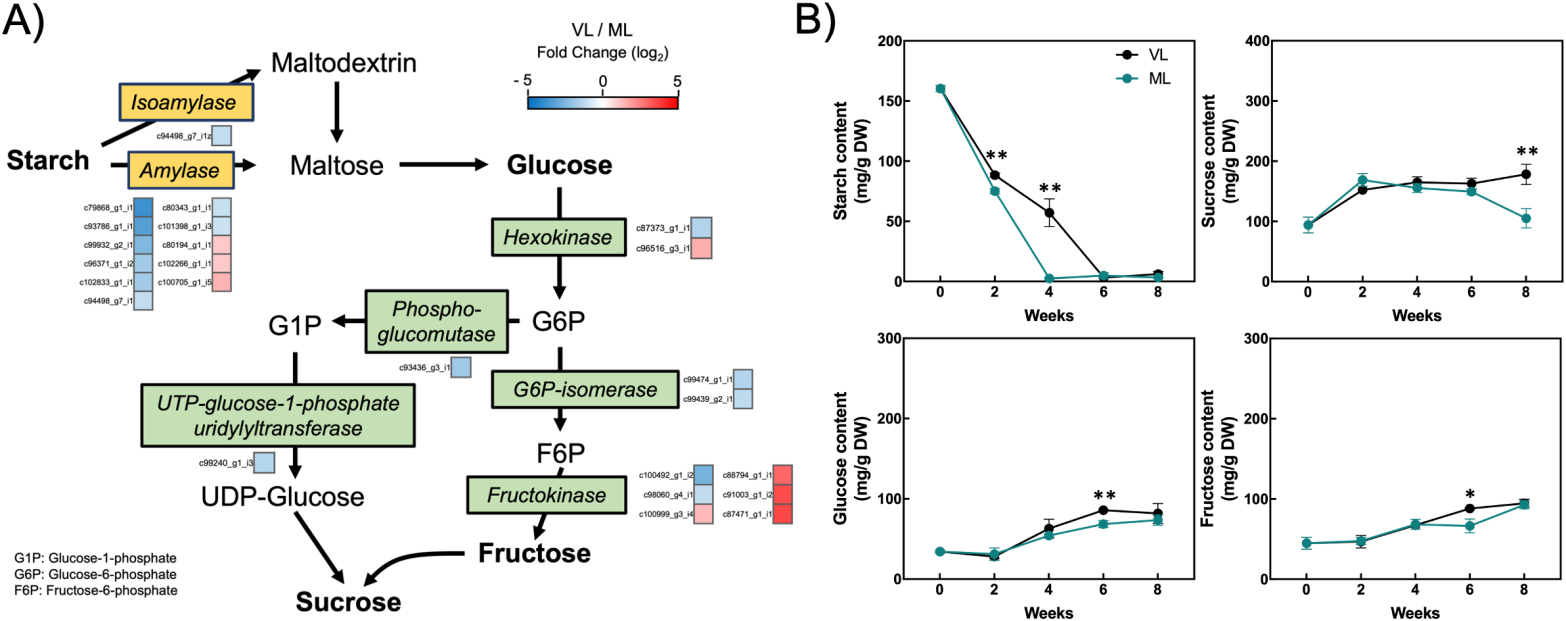
Starch degradation pathway. **(A)** Differentially expressed genes (colored squares below or to the right of each gene) with absolute log_2_ fold change (|log_2_ FC| ≥ 1), p < 0.05, and false discovery rate (FDR) < 0.05. **(B)** Changes in the starch, sucrose, glucose, and fructose content in hardy kiwifruit during storage. Asterisks indicate significant differences between the VL and ML conditions, according to Student’s *t*-test (*p < 0.05 and **p < 0.01; *n* = 3).

The accumulation of soluble carbohydrates in fruits is closely associated with chilling tolerance during cold storage, as well as with the overall fruit quality (Der Agopian et al., 2011; Cao et al., 2013). We examined the changes in unigene expression related to starch degradation and sucrose biosynthesis, as identified among the DEGs in the RNA-seq dataset. Hardy kiwifruit contain high levels of starch at commercial harvest, which gradually degrades into soluble sugars during storage as the fruit ripens. Most unigenes encoding amylase enzymes were downregulated under the VL conditions compared with that under the ML conditions (**Figure 9A**; **Supplementary Table S5**). Similarly, unigenes associated with *glucose-6-phosphate isomerase*, *phosphoglucomutase*, and *UTP-glucose-1-phosphate uridylyltransferase* exhibited lower expression in the VL group than in the ML group. In contrast, several unigenes encoding hexokinase and fructokinase were significantly upregulated under the VL conditions. To elucidate the primary metabolic responses to cold storage, we quantified the levels of starch and soluble sugars (glucose, fructose, and sucrose) during storage (**Figure 9B**). In the ML group, starch levels decreased rapidly during the first 4 weeks, showing a significant difference compared to the VL group. In contrast, starch degradation in the VL group proceeded more slowly, extending over a 6-week period. This indicated that starch breakdown closely coincided with fruit ripening. Interestingly, sucrose levels were higher in the VL group than in the ML group after 2 weeks, although the difference was statistically significant only at 8 weeks. Moreover, glucose and fructose concentrations were significantly higher in the VL samples than in the ML samples at 6 weeks of storage. These findings indicate that storage temperature influences soluble sugar levels in hardy kiwifruit, with lower temperatures favoring their accumulation during the later phase of cold storage.

## 4 Discussion

### 4.1 Characteristics of chilling injury in hardy kiwifruit

Cold storage systems are essential for maintaining the quality and extending the shelf life of fresh fruits and vegetables, and are widely used in commercial agriculture as well as in the food industry. However, different fruit and vegetable cultivars exhibit varying degrees of sensitivity to cold-storage conditions. Exposure to inappropriately low-temperature environments can result in chilling injury, which significantly compromises the quality of fruits and their marketability (Wang, 1989; Lurie and Crisosto, 2005; Patel et al., 2016). Therefore, a thorough understanding of the specific cold response mechanisms in each fruit type is critical for optimizing storage conditions and minimizing postharvest losses. Fruits and their cultivars exhibit a variety of visible physiological disorders in response to cold-storage conditions (Wang, 1989). For instance, peaches often develop physiological disorders, such as dry flesh, browning of the flesh or pit cavity, and internal reddening when stored at 2–5°C for 1 to 2 weeks; however, development of these symptoms is delayed when the fruit is stored at 0°C (Lurie and Crisosto, 2005). Mango fruit exhibits cultivar-dependent susceptibility to cold storage, with cold-sensitive cultivars developing peel browning earlier than cold-tolerant ones when stored at 4°C (Chongchatuporn et al., 2013). In hardy kiwifruit, cold conditions often lead to peel browning and pitting during storage (Eo et al., 2024; Park et al., 2024). Such damage is particularly evident in specific cultivars when hardy kiwifruit is stored near the freezing point. In this study, we compared the physiological differences and visible chilling injury in hardy kiwifruit stored under VL and ML conditions. Fruit ripening was significantly delayed under VL conditions relative to that under ML conditions, as indicated by higher fruit firmness and lower weight loss over the eight-week storage period (**Figure 1**). However, peel pitting, a key symptom of chilling injury in hardy kiwifruit, was substantially more pronounced under VL conditions as storage progressed (**Figure 2**). These results are consistent with those of previous reports, indicating that the cold storage conditions used in this study are suitable for dissecting the differential cold responses between storage temperatures. In addition, while VL storage effectively delays ripening to prolong shelf life, it also exacerbates cold injury symptoms over time, indicating a trade-off between ripening control and susceptibility to chilling injury at lower temperatures. Various preservation techniques and pretreatment strategies have been explored to mitigate chilling injury symptoms during cold storage across different fruit species and cultivars (Zhang et al., 2021). Low-temperature conditioning, either alone or in combination with phytohormone treatment, is effective in reducing chilling injury (Cai et al., 2006; Yang et al., 2013; Shi et al., 2019). These approaches harness the intrinsic ability of the fruit to acclimate to cold environments, often through a priming effect triggered by the treatment. Such strategies may also serve as effective tools for reducing chilling injury in hardy kiwifruit while simultaneously maintaining fruit freshness during cold storage, although further investigation is needed to confirm their efficacy.

### 4.2 Relationship between the plant antioxidant system and chilling injury in kiwifruit

The plant antioxidant system is a critical defense mechanism that mitigates the oxidative damage caused by ROS generated during internal physiological processes, such as photosynthesis and respiration, and by external environmental stressors, including drought, high and low temperatures, and nutrient deficiency (Tripathy and Oelmüller, 2012; Dumont and Rivoal, 2019). Activation of the antioxidant system typically involves not only the upregulation of the activities of antioxidant enzymes but also the biosynthesis of antioxidant compounds (Castro et al., 2021; Hasanuzzaman and Fujita, 2022; Qian et al., 2024). This system plays a pivotal role in plant responses to cold-induced oxidative damage, including the prevention of plasma membrane deterioration caused by lipid peroxidation following the accumulation of ROS in inter- and intracellular spaces (Airaki et al., 2012; Dreyer and Dietz, 2018; Manasa S et al., 2022). Cold-induced oxidative damage also occurs in postharvest fruits stored under suboptimal low-temperature conditions, and the severity of internal and external chilling injury is influenced by the storage temperature and fruit cultivar (Brizzolara et al., 2018; Eo et al., 2024; Kamble et al., 2024; Park et al., 2025). Several reports have shown that the antioxidant system plays critical roles in maintaining fruit integrity in the cold-storage environment. The mango fruit were reported to manifest different cold susceptibility between two different cultivars, wherein cold-susceptible cultivar exhibited early peel browning compared with cold-tolerant cultivar with differential SOD and catalase (CAT) activities between storage at 4°C and 12°C, whereas cold-tolerant cultivar showed comparable enzyme activities between the temperatures (Chongchatuporn et al., 2013). Postharvest kiwifruit treated with γ-aminobutyric acid enhance chilling tolerance in which activation of AsA biosynthesis and accumulation partly contribute to alleviate chilling-induced oxidative damages (Liu et al., 2023). Likewise, storage conditions maintaining higher activities of antioxidant enzymes, such as SOD, CAT, and APX, in mume fruit showed lower chilling injury during the storage period (Imahori et al., 2008). In addition, low-temperature conditioning of fuzzy kiwifruit enhanced the activities of antioxidant enzymes, such as SOD, CAT, APX, and POD, leading to the reduction of cold injury symptoms via inhibition of superoxide radicals and hydrogen peroxide accumulation (Yang et al., 2013). Similarly, GST plays a critical role in alleviating cold-induced oxidative damage in plants, particularly by reducing organic hydroperoxides of fatty and nucleic acids to their corresponding monohydroxy alcohols (Dixon et al., 2002; Yang et al., 2016; Song et al., 2021; Ding et al., 2022). Additionally, the role of GST in chilling tolerance of fruits has been demonstrated in previous reports. MabHLHs, as transcription factors related to the expression of the GST family, were downregulated under cold stress in banana, leading to chilling injury in the banana peel (Lin et al., 2024). The reduction in chilling injury in peaches induced by γ-aminobutyric acid treatment was reported to coincide with the upregulation of GST activity in peach fruit (Yang et al., 2011). Taken together, the antioxidant and detoxification systems are closely associated with chilling tolerance of fruits during cold storage. In the present study, severe cold injury symptoms such as pitting were markedly observed in hardy kiwifruit stored under the VL conditions (**Figures 2**, **3**). Additionally, continuous accumulation of MDA was detected in the VL group during storage (**Figure 6**), indicating that VL conditions induce cold-related oxidative damage in hardy kiwifruit. Consequently, fruit stored under VL conditions experienced more severe chilling injury than those stored under ML conditions. To counteract this stress, the majority of unigenes associated with antioxidant activity and detoxification were upregulated in fruit stored under VL conditions compared with that in fruit stored under ML conditions (**Figure 7**), suggesting an active response in alleviating cold-induced oxidative damage. Furthermore, hardy kiwifruit stored under VL conditions exhibited higher levels of nonspecific antioxidant compounds, as evidenced by elevated DPPH- and ABTS-radical scavenging activities (**Figure 6**), which likely contributed to the mitigation of ROS accumulation under cold stress. In contrast, the AsA content in the VL group fruit was significantly lower than that in the ML group ones, and this decrease coincided with the downregulation of *GalLDH* and *GPP*, which are involved in AsA biosynthesis, as well as *DHAR*, *MDHAR*, and *APX*, which are involved in regeneration pathway (**Figures 6**, **8B**). In the peel of mango fruit, AsA concentration declined sharply during the early phase of storage at 4°C, accompanied by a relatively high chilling injury index, although it increased during the later phase compared with fruit stored at 12°C (Chongchatuporn et al., 2013). In contrast, AsA levels in pear and peach steadily decreased with increasing chilling injury index; however, this decline was alleviated by treatments with plant-derived compounds such as γ-aminobutyric acid and melatonin, which maintained higher AsA concentrations and reduced chilling injury severity (Zhou et al., 2022; Liu et al., 2024). These findings indicate that changes in AsA levels during cold storage reflect the severity of chilling injury, depending on fruit type and storage duration. Therefore, in the present study, it can be inferred that maintaining higher AsA levels is crucial for mitigating cold-induced oxidative stress, and that the absence of upregulation in AsA biosynthetic and regenerative pathways is a key factor contributing to the decrease in AsA levels when it is utilized as a primary antioxidant. Additionally, *GSTs* were significantly upregulated under the VL conditions at 4 weeks of storage (**Figure 7**), indicating that *GSTs* are key cold-responsive genes in hardy kiwifruit and may contribute to recovery from oxidative damage during cold storage. In the present study, although the expression of AsA biosynthesis- and regeneration-related genes in ‘Autumn Sense’ was not strongly activated under VL conditions, it is possible that AsA-related metabolic changes are influenced by cultivar-specific properties. In previous research, AsA levels in ‘Daebo’ did not decrease at lower storage temperatures, whereas ‘Cheongsan’ showed a similar trait to ‘Autumn Sense’ during cold storage (Eo et al., 2024). Therefore, further investigation of cultivar-specific cold responses of AsA metabolism–related genes and associated antioxidant enzyme activities, such as SOD, POD, APX, and GST, may help clarify their precise molecular and biochemical functions in the cold stress response of hardy kiwifruit, and reinforce the notion that activation of antioxidant and detoxification systems are critical for preserving fruit quality under cold storage conditions.

### 4.3 Carbohydrate accumulation in hardy kiwifruit in response to cold environment

Carbohydrates, including glucose, fructose, and sucrose, play essential roles in plant growth, development, structural integrity, energy supply, and overall metabolic function. In addition, carbohydrate levels in plants respond dynamically to environmental conditions such as low temperatures (Kontunen-Soppela et al., 2002; Zhou et al., 2020; Orzechowski et al., 2021). Although the precise functions of individual carbohydrates under low-temperature conditions are yet to be fully elucidated, they are believed to play a key role in maintaining the integrity of plant cells by acting as osmoregulators and cryoprotectants (Ruelland et al., 2009). Hardy kiwifruit exhibited a sharp decrease in starch levels during the first two weeks of storage under both VL and ML conditions (**Figure 9B**), although a significant difference in starch content was observed between the treatments. This suggests that the initial starch breakdown during cold storage was relatively similar between the VL and ML conditions. However, starch levels showed a marked difference between treatments at 4 weeks, which corresponded with the downregulation of most genes associated with starch degradation under VL conditions compared with that under ML conditions (**Figure 9A**). Nevertheless, the levels of soluble sugars, such as sucrose, glucose, and fructose, under VL conditions were slightly higher than or comparable to those under ML conditions at 4 weeks. Moreover, at 6 weeks, the levels of these sugars, particularly sucrose, were significantly higher in the VL group fruit than in those stored under ML conditions. Although genes encoding sucrose synthase, a key enzyme in sucrose biosynthesis, were not included among the DEGs in our transcriptomic data, a subset of hexokinase and fructokinase genes was upregulated under VL conditions compared with that under ML conditions (**Figure 9A**). These results are consistent with previous findings in other hardy kiwifruit cultivars (Eo et al., 2024). Cold-responsive changes in carbohydrate content have been previously documented for a variety of fruit cultivars. The content of soluble carbohydrates and polyols was reported to be consistently higher in zucchini fruit stored at 1°C compared with the content at 4°C throughout the storage period (Palma et al., 2014). In addition, sucrose levels were significantly higher, whereas glucose and fructose levels were lower, in peach fruit stored at 0°C compared with that in fruit stored at 5°C (Wang et al., 2013). Cao et al. (2013) investigated the differences in soluble carbohydrate content between chilling injury-tolerant and -susceptible loquat cultivars, and found that the tolerant cultivar contained higher levels of glucose and fructose than the susceptible cultivar when stored at 1°C. These studies support the notion that specific soluble carbohydrates accumulate more during cold storage at lower temperatures. Furthermore, pairwise Pearson’s correlation coefficient analysis revealed a statistically significant positive correlation between pitting and soluble sugars, such as glucose and fructose (**Supplementary Figure S2**), suggesting that accumulated soluble sugars may be associated with the mitigation of cellular damage in hardy kiwifruit during cold storage. Moreover, it can be speculated that slower degradation of starch contributes to the retention of soluble sugars during storage at relatively lower temperatures. Although delayed ripening of hardy kiwifruit under VL conditions is one reason for reduced starch degradation during storage, further investigation into the specific role of starch degradation in chilling injury will provide valuable insights into the mechanisms underlying the cold response in hardy kiwifruit.

## 5 Conclusion

Cold storage is widely employed as an effective method for maintaining the freshness of hardy kiwifruit. However, a major limitation of this approach is the high susceptibility of the fruit to chilling injury, especially when stored at low or near-freezing temperatures. In the present study, we investigated the physiological and molecular responses of hardy kiwifruit stored at low temperatures. Our findings indicate that activation of the antioxidant system and carbohydrate accumulation, associated with delayed starch degradation, are induced under VL conditions, potentially contributing to the alleviation of chilling injury. Nevertheless, AsA biosynthetic pathway were not strongly activated under VL conditions, and AsA concentrations declined sharply relative to ML conditions. These results, however, suggest that AsA may function as a major antioxidant in mitigating oxidative damage associated with chilling injury. Consequently, if storage conditions can be optimized to stimulate the antioxidant system in fruit, the incidence of chilling injury may be significantly reduced during long-term near-freezing storage while maintaining fruit freshness. Further investigations are required to identify effective physical or chemical treatments capable of enhancing these antioxidant mechanisms, thereby providing promising strategies for preserving fruit quality during prolonged cold storage.

## Data Availability

The datasets presented in this study can be found in online repositories. The names of the repository/repositories and accession number(s) can be found below: https://www.ncbi.nlm.nih.gov/, PRJNA1028359.
