# Supplementary material for: Cold storage characteristics of hardy kiwifruit, Actinidia arguta ‘Autumn Sense’: comparison between two cold storage temperatures
Source: Front Plant Sci. 2025 Oct 22;16:1692735. doi: 10.3389/fpls.2025.1692735 (PMC12586017; doi:10.3389/fpls.2025.1692735)
Supplement: Supplementary file 1 [file DataSheet1.pdf]

SUPPLEMENTARY MATERIAL

TITLE: Cold storage characteristics of hardy kiwifruit, *Actinidia arguta* 'Autumn Sense': comparison between two cold storage temperatures

AUTHOR INFORMATION:

AUTHORS

Uk Lee, Hyun Ji Eo, Chung Ryul Jung, and Yonghyun Kim*

AUTHOR AFFILIATIONS

Special Forest Resources Division, National Institute of Forest Science, Gwonseon-gu, Suwon 16631, Republic of Korea

***CORRESPONDING AUTHOR**

yonghyun24@korea.kr

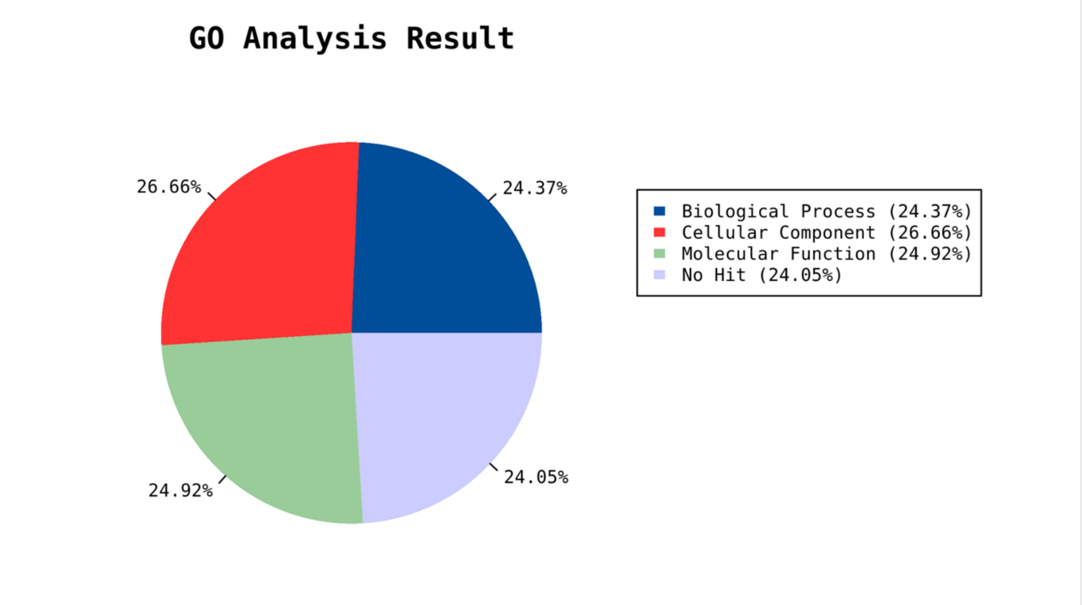

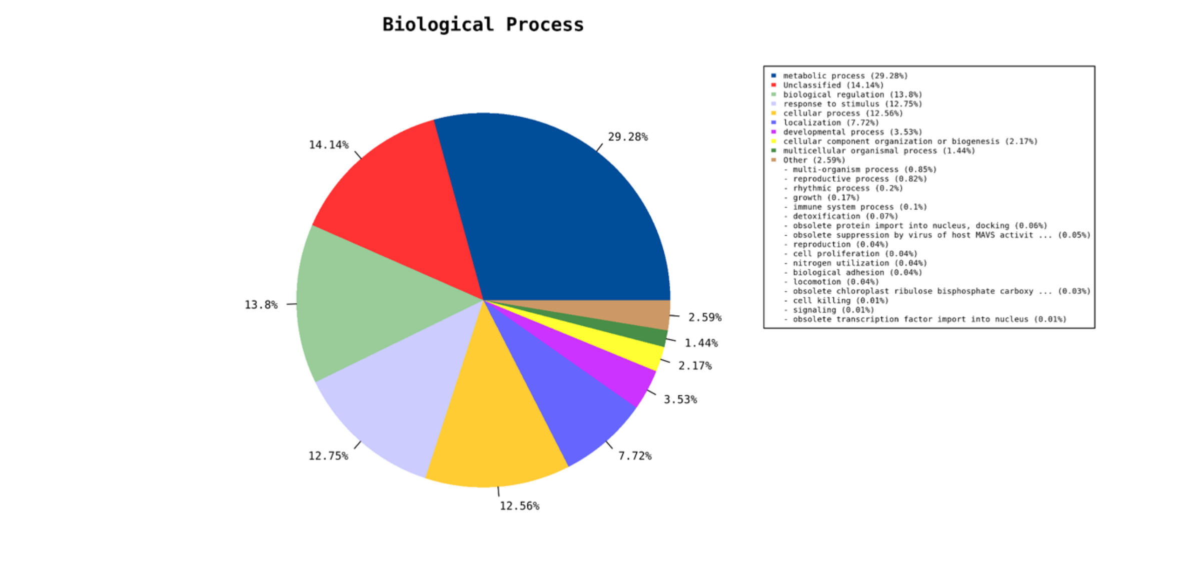

Figure S1. The gene ontology (GO) database was used for classifying the annotated unigenes employing the BLASTX tool in DIAMOND. The resulting GO terms were categorized into three main domains: biological process (BP), cellular component (CC), and molecular function (MF).

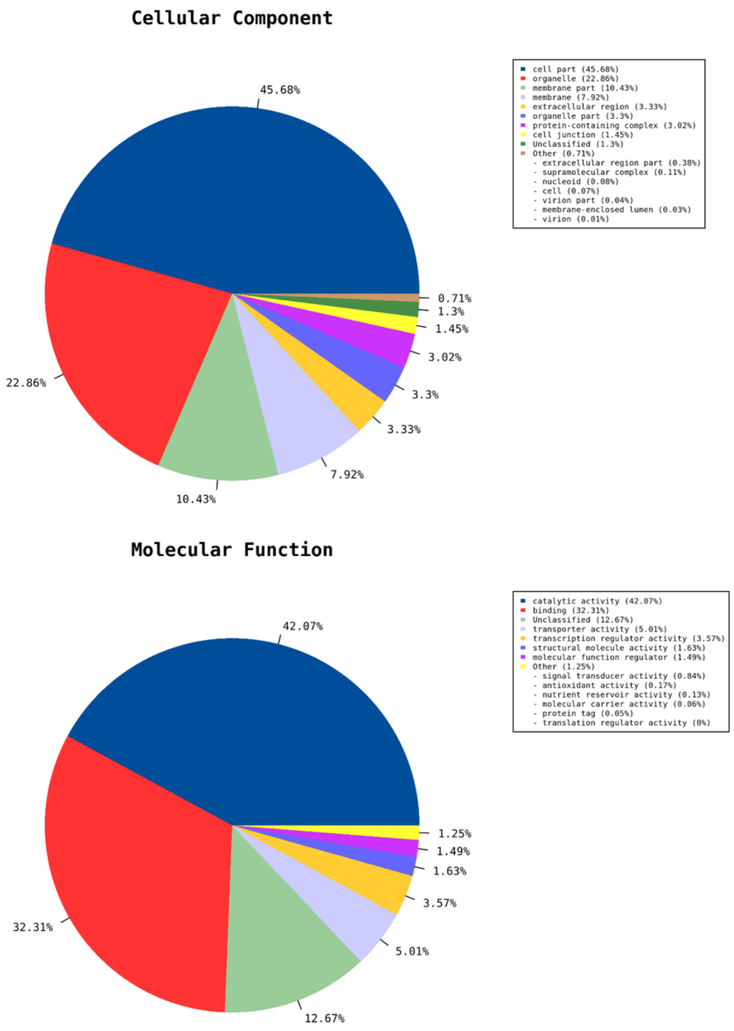

Figure S1. Continued

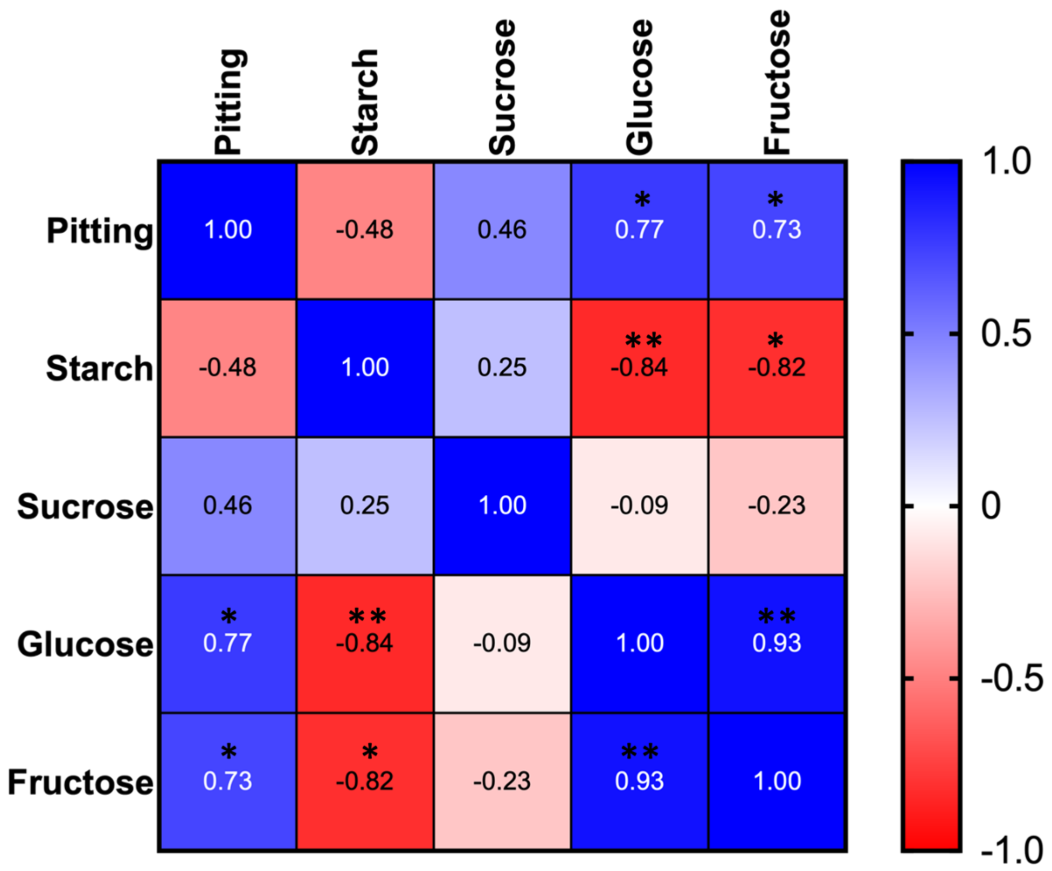

Figure S2. A pairwise Pearson's correlation coefficient matrix (values in boxes) was generated in GraphPad Prism version 10 (GraphPad Software, San Diego, CA, USA) to show the relationship between the chilling injury symptom (pitting) and carbohydrate levels. The color scale represents the strength of the correlation, and asterisks indicate statistical significance (*p < 0.05, **p < 0.01).

Table S1. List of primers used for RT-qPCR analysis.

Gene		Primer Sequence (5'-3')
AaDHAR	Forward	GCCGCTTCTGGTTCTCCTGAT
(Lin et al., 2022)	Reverse	TCGGAGTCAGGAATCCATTTGTC
AaMDHAR	Forward	TAGAAGCAGACACGATTG
(Lin et al., 2022)	Reverse	ACCATCAACCTGTATTCC
AaAPX	Forward	TTGTTCCGTCCATATTGA
(Lin et al., 2022)	Reverse	TTATTCTCTGGTCTGCTAAT
AaActin	Forward	GCTTACAGAGGCACCACTCAACC
(Lin et al., 2022)	Reverse	CCGGAATCCAGCACAATACCAG
AaGalDH	Forward	GATTGTGAATGAGACGATT
(Lin et al., 2022)	Reverse	GGAGTCCTGTAATACCAA
AaGalLDH	Forward	TCCTATTGACGAGCAAGT
(Lin et al., 2022)	Reverse	AGCGAGCAAGATAGAAGA
AaGPP	Forward	TGTGACGATTGCTGATAA
(Lin et al., 2022)	Reverse	ACCATTCTCCTCTCCATA
AaGalUR	Forward	GATGCCGGACCTGGAAACT
(Lin et al., 2022)	Reverse	ACTTGATGATGCCGAGATGG
c90830_g2_i1_*SOD*	Forward	ACTGTGGAAGAGCTGAGAA
	Reverse	GCGGGGAAAGGAAGAGAA
c70277_g1_i1_*GST1*	Forward	CCCGACAGAGAGAAATTG
	Reverse	CAAAACACCTGCAAATCAC
c94690_g1_i3_*GST2*	Forward	AGAGTGGCGAAAGGAAAA
	Reverse	GCAACCACATCCCAAAAA

References

Lin et al., 2022, L-ascorbic acid metabolism in two contracting hardy kiwifruit (*Actinidia arguta*) cultivars during fruit development, Scientia Horticulturae 297:110940
